# Expression of prostasin and its inhibitors during colorectal cancer carcinogenesis

**DOI:** 10.1186/1471-2407-9-201

**Published:** 2009-06-25

**Authors:** Joanna Selzer-Plon, Jette Bornholdt, Stine Friis, Hanne C Bisgaard, Inger MB Lothe, Kjell M Tveit, Elin H Kure, Ulla Vogel, Lotte K Vogel

**Affiliations:** 1 Department of Cellular and Molecular Medicine, Faculty of Health Science, University of Copenhagen, Denmark; 2 Department of Pathology, Ulleval University Hospital, Oslo, Norway; 3 The Cancer Centre, Ulleval University Hospital, 0407 Oslo, Norway; 4 Department of Environmental and Health Studies, Telemark University College, Bø, Norway; 5 National Food Institute, Technical University of Denmark, Soborg, Denmark and the National Research Centre for the Working Environment, Copenhagen, Denmark

## Abstract

**Background:**

Clinical trials where cancer patients were treated with protease inhibitors have suggested that the serine protease, prostasin, may act as a tumour suppressor. Prostasin is proteolytically activated by the serine protease, matriptase, which has a very high oncogenic potential. Prostasin is inhibited by protease nexin-1 (PN-1) and the two isoforms encoded by the mRNA splice variants of *hepatocyte growth factor activator inhibitor-1 *(*HAI-1*), *HAI-1A*, and *HAI-1B*.

**Methods:**

Using quantitative RT-PCR, we have determined the mRNA levels for *prostasin *and *PN-1 *in colorectal cancer tissue (n = 116), severe dysplasia (n = 13), mild/moderate dysplasia (n = 93), and in normal tissue from the same individuals. In addition, corresponding tissues were examined from healthy volunteers (n = 23). A part of the cohort was further analysed for the mRNA levels of the two variants of HAI-1, here denoted *HAI-1A *and *HAI-1B*. mRNA levels were normalised to *β-actin*. Immunohistochemical analysis of prostasin and HAI-1 was performed on normal and cancer tissue.

**Results:**

The mRNA level of prostasin was slightly but significantly decreased in both mild/moderate dysplasia (p < 0.001) and severe dysplasia (p < 0.01) and in carcinomas (p < 0.05) compared to normal tissue from the same individual. The mRNA level of *PN-1 *was more that two-fold elevated in colorectal cancer tissue as compared to healthy individuals (p < 0.001) and elevated in both mild/moderate dysplasia (p < 0.01), severe dysplasia (p < 0.05) and in colorectal cancer tissue (p < 0.001) as compared to normal tissue from the same individual. The mRNA levels of *HAI-1A *and *HAI-1B *mRNAs showed the same patterns of expression. Immunohistochemistry showed that prostasin is located mainly on the apical plasma membrane in normal colorectal tissue. A large variation was found in the degree of polarization of prostasin in colorectal cancer tissue.

**Conclusion:**

These results show that the mRNA level of *PN-1 *is significantly elevated in colorectal cancer tissue. Future studies are required to clarify whether down-regulation of prostasin activity via up regulation of PN-1 is causing the malignant progression or if it is a consequence of it.

## Background

Extracellular proteases and protease inhibitors are believed to play an important role during carcinogenesis in many different ways such as degrading the extracellular matrix in order to facilitate invasive growth and activating signal molecules. In accordance with this, proteases were mostly thought of as promoters of carcinogenesis. However, clinical trials where cancer patients were treated with broad-range protease inhibitors have shown that proteases can act as tumour suppressors [1]. Studies using loss-of-function animal models have further confirmed the existence of extra-cellular proteases with anti-tumour properties [2-4]. Prostasin is a relatively unknown extracellular serine protease suspected to play a role as tumour suppressor [1].

Prostasin is a glycosylphosphatidylinositol (GPI)-anchored trypsin-like protease expressed in most epithelial cells [5,6]. It is to some degree shed from the membrane and found as a soluble enzyme [7]. The proteolytical activity of prostasin can be inhibited by protease nexin-1 (PN-1) [8,9] also known as glia-derived nexin (GDN) or serpin E2 [10] and by the two isoforms of hepatocyte growth factor activator inhibitor-1 (HAI-1), probably originating from two mRNA splice variants of *HAI-1*, here denoted *HAI-1A*, and *HAI-1B *[11]. HAI-1B differs from HAI-1A by a 16-amino acid insertion [12]. Prostasin is part of the matriptase-prostasin proteolytic cascade regulating terminal epidermal differentiation [13]. Matriptase is thought to be the first protease in the cascade due to its ability to auto-activate [14,15] and because prostasin is activated by a matriptase-catalysed cleavage [13]. The downstream target for prostasin is unclear but the matriptase-prostasin cascade eventually regulate the processing of the differentiation marker filaggrin [16,17] and is essential for establishment of epidermal integrity [17,18]. It has furthermore been shown that prostasin can activate the epithelial sodium channel (ENaC) [19] and cleave the epidermal growth factor receptor [20] but may very well also have other substrates. Matriptase and prostasin share the same inhibitors, as both are inhibited by HAI-1A, HAI-1B [11,12], and PN-1 [21]. Using transgenic mice it has been shown that deregulated matriptase causes carcinogenesis. Even a modest over-expression of matriptase in the skin of transgenic mice caused spontaneous squamous cell carcinoma in 70% of the mice [22]. Simultaneous over-expression of the matriptase inhibitor HAI-1 completely negated the oncogenic effects of matriptase [22]. Although not known it is unlikely that the oncogenic properties of matriptase are exerted via activation of prostasin as prostasin over-expression has been shown to cause reduced *in vitro *invasiveness in both prostate and mammary cancer cell lines [23,24] and high *prostasin *mRNA levels correlates with longer survival for gastric cancer patients [25].

Prostasin is expressed in most epithelial cells and could potentially play a role in most types of cancer. Prostasin has been shown to be down-regulated in gastric cancers [25] and prostate cancer [26]. The enzymatic activity of prostasin may be regulated by several mechanisms one of them being by regulating the mRNA expression levels of its inhibitors PN-1, HAI-1A, and HAI-1B. Elevated PN-1 levels have been found in pancreatic, breast and oral cancers [27-29]. PN-1 does not seem to be a specific inhibitor for prostasin and matriptase as it also inhibits thrombin, trypsin, plasmin and plasminogen activators [30-32] and functions as a neurite growth promoting factor in neuroblastoma cells [33]. HAI-1A and HAI-1B are also non specific inhibitors for prostasin and matriptase as they also inhibit hepatocyte growth factor activator and trypsin [12]. No physiological difference between HAI-1A and HAI-1B has been found. We have previously observed that the *HAI-1 *mRNA levels are decreased during colorectal cancer carcinogenesis in both normal and affected tissue from individuals with colorectal cancer adenomas and carcinomas as compared to healthy individuals using an assay that does not differentiate between *HAI-1A *and *HAI-1B *[34]. Likewise HAI-1 has been shown to be down-regulated in renal cell carcinomas [35] and in gastric and colorectal cancer [36].

In the present study we have investigated the mRNA levels of *prostasin *and its inhibitors *PN-1*, *HAI-1A*, *and HAI-1B*, during colorectal cancer carcinogenesis in humans.

## Methods

### Subject population

The KAM cohort (Kolorektal cancer, Arv og Miljø) is based on the screening group of the Norwegian Colorectal Cancer Prevention study (the NORCCAP study) in the county of Telemark, Norway [37]. Additionally, a series of colorectal cancer cases were recruited to the KAM cohort from routine clinical work at Telemark Hospital in Skien and Ulleval University Hospital in Oslo. The ID number for the NORCCAP study at Clinicaltrials.gov is NCT00119912 [38]. In the NORCCAP study a total of 20,780 men and women, age distribution 50–64 years, drawn randomly from the population registries in Oslo (urban) and the county of Telemark (mixed urban and rural) were invited to have a flexible sigmoidoscopy (FS) screening examination with or without (1:1) an additional faecal occult blood test (FOBT). 777 (4%) individuals were excluded according to exclusion criteria [37]. The KAM biobank currently consists of 234 colorectal cancer cases, 1044 cases with adenomas (229 high-risk, 762 low-risk and 53 hyperplastic polyps) and 400 controls. Controls were defined as individuals with normal findings at the time of FS. The KAM study is approved by the Regional Committee for Medical Research Ethics and the Norwegian Data Inspectorate. In the present study we have analyzed cases with carcinoma (n = 116), cases with adenoma (n = 106) and controls that were polyp-free at time of FS (n = 23). Each case was classified according to the degree of malignancy in the lesion. For individuals with adenoma a sample of control tissue was collected 30 cm from the anus. Control samples from healthy individuals were taken from individuals, where no adenomas or carcinomas could be identified with FS. The histology of the adenomas was examined independently by two histopathologists to categorize the degree of dysplasia as either mild/moderate (n = 93) or severe (n = 13). They reached consensus in all cases. Cases of dysplasia were also classified as either low- or high-risk according to the size and/or differentiation state of the adenoma. A high-risk adenoma is defined as an adenoma measuring more than 10 mm in diameter and/or with villous components or showing severe dysplasia. [37]. The distribution of gender and age among controls and cases with carcinoma or adenoma of the colon is shown in Table 1.

**Table 1:** Characteristics of cases and healthy individuals participating in this study

Prostasin	Healthy	Cases		
		Adenomas^1^		Carcinomas

		Mild/moderate dysplasia	Severe dysplasia	

	(n = 23)	(n = 93)	(n = 13)	(n = 116)

**Men**	9	68	7	59

**Women**	14	25	6	57

**Mean age +SD**^2^	56.3 ± 4.6	57.3 ± 3.5	54.8 ± 3.1	68.7 ± 12.4

^1^The adenomas are divided in groups according to diagnosed degree of dysplasia.

^2^There are significant differences in age among the four groups of healthy and affected individuals at 95% confidence level (Kruskal-Wallis test).

### RT-PCR

Total RNA was purified from tissue as recommended by the manufacturers using E.Z.N.A. Total RNA Kit II (VWR, Copenhagen, Denmark). The tissue samples were frozen in liquid as soon as possible after surgery and were stored in liquid N_2 _until RNA purification. RNA purification included a DNAse treatment. cDNA synthesis was performed on approximately 200 ng RNA per 20 μl using High-Capacity cDNA Archive Kit (Applied Biosystems, Denmark). Quantitative RT-PCR for *prostasin *was performed on the ABI7300 sequence detection system (Applied Biosystems) in Universal Mastermix (Applied Biosystems) using 160/100 nM probe and 600/200 nM primers for *prostasin *and *PN-1*, respectively. For detection of *prostasin *mRNA, primers and probes were: F, 5' GGCCTCCACTGCACTGTCA 3'; R,5' AGTTACACGTCTCACGACTGATCAG '3'; probe, 5'-FAM-CAGTGAGCCTCCTGACGCCCAAG-BHQ-3'. For detection of *PN-1 *mRNA, primer and probes were: F, 5' CCTCCATGTTTCTCATATCTTGCA 3'; R, 5' ACCAGGGAGGCGATGATCT 3'; probe, 5'FAM-AAGCTTCAGCAGCAACAACTGCAATTCTCA-BHQ-3'. Primers were designed within different exons and with probes covering the exon-exon border to prevent amplification of genomic DNA. DNA coding for *HAI-1 *gives rise to two mRNA's coding for two proteins where the amino acids sequence differs by 16 residues [12]. We have previously measured the *HAI-1 *mRNA using a probe/primer set that detects both *HAI-1A *and *HAI-1B*. For this study we have designed new sets of probe/primers specific for either *HAI-1A *or *HAI-1B*. Assays for *HAI-1A *and *HAI-1B *were performed using 300 nM probe and 100 nM primers. Primers and probes for detection of *HAI-1A *were: F, 5'AGA TCT GCA AGA GTT TCG TTT ATG G-3'; R, 5'-GGT GCC AGA GCA CAC TGG AT-3'; probe, 5'-FAM-TGT GCA AGG CCC CTC CAT GGA A-BHQ-1-3'. Primers and probes for detection of *HAI-1B *were: F, 5'-ATC TGC AAG AGT TTC GTT TAT GGA-3'; R, 5'-GGT GCC AGA GCA CAC TGG AT-3'; probe, FAM-CCT TTG AGA GGC AGC TC-MGBNFQ. *HAI-1A *and *HAI-1B *probes were obtained from Applied Biosystems. *β-actin *primers and probes were obtained as a pre-developed assay (cat. no. 4310881E). All other primers and probes were obtained from TAG Copenhagen (Denmark). In a validation experiment using a control sample, a dilution series was produced and assayed for *prostasin*, *PN-1*, *HAI-1A*, *HAI-1B *and *β-actin *as described for the comparative C_t _method [39]. When C_t _values were plotted against log dilution it was shown that the assays are quantitative over a range of 256-fold dilution and that the PCR reactions have similar efficiencies provided that a threshold of 0.02 is used for prostasin and 0.2 is used for *PN-1*, *HAI-1A*, *HAI-1B*, and *β-actin*. The threshold is a fixed fluorescence signal level above the baseline and the C_t _value of a sample is determined as the fractional cycle number where the sample's fluorescence signal exceeds the threshold. *Prostasin*, *PN-1*, *HAI-1A*, *HAI-1B*, and *β-actin *were quantified separately in triplicates. The average standard deviation on the triplicates was 14% or less. The standard deviation on repeated measurements of the same sample (the control) in separate experiments was 20%, 11%, 14%, and 14% for *prostasin*, *PN-1*, *HAI-1A *and *HAI-1B *respectively, indicating the day-to-day variation of the assay. Independent PCR reactions of the same samples yielded a correlation coefficient of 0.85 for *prostasin *and 0.95 for *PN-1*. Negative controls (where the RNA was not converted into cDNA) and positive controls were included in all sets.

### Statistical analysis

MiniTab Statistical Software, Release 13.1 Xtra (Minitab Inc.) and Graph Pad Prism 4 were used for the statistic calculations. The data were not adjusted for gender since the incidence ratio of colorectal cancer between the genders is 1:1 in Norway [40].

### Immunohistochemistry

Tissue specimens were fixed in 4% formalin, paraffin embedded and processed for routine histology. Tissue sections were cleared with xylene and rehydrated in a graded series of alcohols and boiled in T-EG buffer (10 mM Tris, 0.5 mM EDTA, pH = 9) for 20 min for antigen retrieval. The sections were blocked for 30 min in PBS containing 3% BSA and 0.1% Triton X-100 and then incubated at 4°C overnight with mouse anti-prostasin antibody (BD Biosciences, cat nr. 612173) (0.02 μg/μl) and goat anti-hHAI-1 (RD System, cat nr. AF1048, diluted 1:1000). The slides were washed 3 times for 5 min with 3% BSA in PBS and 0.1% Triton X-100 and incubated at room temperature for 45 min with the secondary antibody Alexa fluor 555 donkey anti-mouse and Alexa fluor 488 donkey anti-goat. Afterwards, tissue sections were washed 3 times for 5 min with 3% BSA in PBS and 0.1% Triton X-100, followed by 4 times in PBS and finally mounted with DAPI H-1500 (Vector Laboratories Inc.). The sections were examined for the localization of prostasin subjected to laser conventional fluorescence microscopy. Sections where the primary antibodies were omitted served as controls.

## Results

### Expression of *prostasin *and *PN-1 *mRNA during carcinogenesis

The mRNA levels of prostasin and *PN-1 *were measured in colon tissue samples from healthy individuals (n = 23), individuals with mild/moderate dysplasia (n = 93), individuals with severe dysplasia (n = 13), and individuals with colorectal cancer (n = 116) by real-time RT-PCR. From individuals with dysplasia a sample of histologically normal tissue was used as well. From individuals with colorectal cancer two sample of histologically normal tissue from the surgically removed tissue were used. Ole sample was obtained as far away from the cancer tissue as possible (normal distant) and another sample obtained immediately adjacent to the cancer tissue (normal adjacent). A good correlation between the mRNA and protein levels for both *prostasin *[26] and *PN-1 *[41] has previously been shown. The mRNA levels of *prostasin, PN-1, HAI-1A *and *HAI-1B *were normalised to the mRNA level of *β-actin*.

**Figure 1:**
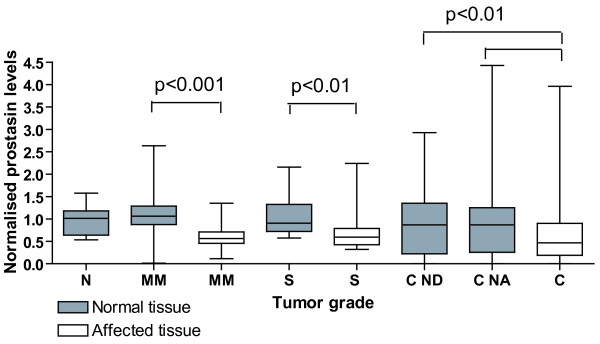
**The levels of *prostasin *mRNA were determined by real-time RT-PCR from healthy individuals (N), individuals with mild/moderate dysplasia (M/M), severe dysplasia (S) or carcinomas (C)**. Both normal (grey bars) and affected (white bars) tissues were examined from each individual. From colorectal cancer patients one sample of histological normal tissue was taken as far away from the colorectal cancer tissue as allowed by the surgically removed tissue (normal distant, here designated ND) and one sample of histological normal tissue was taken immediately adjacent to the colorectal cancer tissue (normal adjacent here designated NA). mRNA levels were normalised to the *β-actin *mRNA level. The box shows the interquartile range, the whiskers the range and the median is indicated by a vertical line.

The range and the interquartile range of mRNA levels of *prostasin *normalised to *β-actin *are shown in Figure 1. When the *prostasin *mRNA level of healthy individuals was compared to normal or affected tissue from individuals with dysplasia or colorectal cancer no significant difference could be observed (Figure 1 and Table 2). However, the *prostasin *mRNA level of all adenomas and carcinomas combined was statistically significantly lower than the level in tissue from healthy individuals (p < 0.05). There was also a significant difference when normal and affected tissue from the same individual were compared for individuals with mild/moderate dysplasia (p < 0.001), severe dysplasia (p < 0.01), and for colorectal cancer both compared to the normal distant (p < 0.05) and to the normal adjacent sample (p < 0.01). This shows that the *prostasin *mRNA level is slightly but significant lower in mild/moderate dysplasia, severe dysplasia and colorectal cancer tissue as compared to normal tissue.

**Table 2:** *Prostasin *mRNA levels in normal and affected tissues.

Variable	mRNA level in normal tissue Mean (SD)	P^a^	mRNA level in adenomas/carcinomas Mean (SD)			P^a^	P^b^
**Prostasin**							

**Healthy individuals**	0.96 (0.29)						

**Mild/moderate**	1.10 (0.38)	NS	0.62 (0.24)			NS	<0.001

**Severe**	1.08 (0.46)	NS	0.73 (0.50)			NS	<0.01

**Carcinoma distant adjacent**	0.85 (0.65)	NS	0.61 (0.60)			NS	<0.05
	0.90 (0.76)	NS					<0.01

**All adenomas and carcinomas**	0.96 (0.61)	NS	0.62 (0.48)			<0.05	ND

**PN-1**							

**Healthy individual**	0.022 (0.030)						

**Mild/moderate**	0.015 (0.017)	NS	0.041 (0.028)			NS	<0.001

**Severe**	0.010 (0.003)	NS	0.033 (0.031)			NS	<0.05

**Carcinoma distant adjacent**	0.028 (0.027)	NS	0.063 (0.060)			<0.001	<0.001
	0.025 (0.018)	NS					<0.001

The mRNA levels were normalised to *β-actin *mRNA levels.

NS = not significant

ND = not determined

p value for the comparison to the expression levels in healthy individuals using a one-way ANOVA and Tukey's post test for paired comparisons.

a) p value for the comparison to the expression levels in healthy individuals using a one-way ANOVA and Tukey's post test for paired comparisons.

b) p value for the comparison of the expression levels in normal and affected tissue from the same individual using a paired t-test

**Figure 2:**
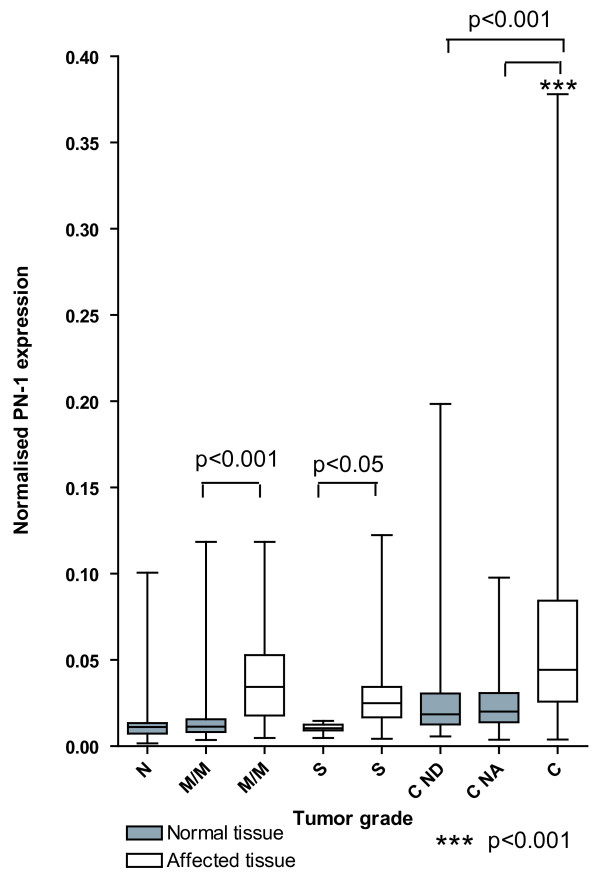
**The levels of *protease nexin-1 *(*PN-1*) mRNA were determined by real-time RT-PCR from healthy individuals (N), individuals with mild/moderate dysplasia (M/M), severe dysplasia (S) or carcinomas (C)**. Symbols and group designations are the same as in Figure 1. mRNA levels were normalised to the *β-actin *mRNA level. The box shows the interquartile range, the whiskers the range and the median is indicated by a vertical line.

The range and the interquartile range of mRNA levels of *PN-1 *normalised to *β-actin *are shown in Figure 2. The *PN-1 *mRNA level showed an increase with increasing tumour grade in dysplastic and cancerous tissues whereas the normal tissue was not affected. A significant almost 3-fold increase (p < 0.001) of *PN-1 *mRNA was found in carcinomas as compared to corresponding tissues in healthy individuals (Table 2 and Figure 1). Comparing the level of *PN-1 *in dysplastic and cancerous tissue with the corresponding normal tissue from the same individual, significant higher levels were encountered in mild/moderate dysplasia (p < 0.001), severe dysplasia (p < 0.05) and in carcinomas both when compared to the normal distant sample and with normal adjacent sample (p < 0.001), (Table 2). In addition we found a significantly higher level of *PN-1 *mRNA when comparing affected tissue from mild/moderate and severe dysplasia combined (all adenomas) with carcinomas (p < 0.001) using a one-way ANOVA and Tukey's post test. The *PN-1 *mRNA levels thus seem to be upregulated both at the transition from normal tissue to mild/moderate dysplasia and then again at the transition between dysplasia and cancer tissue whereas normal tissue is not affected.

Colorectal cancer can be staged according to Dukes' stage A-D where D is the most advanced and metastatic stage. Among the carcinomas in this study 23 carcinomas were designated as Dukes'stage A, 47 carcinomas as stage B, 39 carcinomas as stage C and none of the samples were classified as stage D. No tendency and no significant difference (one-way ANOVA and Tukey's post test for paired comparison) were seen when comparing the *prostasin *and *PN-1 *mRNA levels with the designated Dukes'stages. Likewise the adenomas were classified as either low-risk (n = 14) or high-risk (n = 91) adenomas using the criteria described in material and methods. No significant difference in the mRNA levels of either *prostasin *or *PN-1 *was seen when comparing high-risk and low-risk adenomas (one-way ANOVA and Tukey's post test for paired comparison). When the *prostasin *mRNA level in normal tissue from either healthy individuals or individuals with dysplasia or colorectal cancer was plotted against age we were unable to detect any age-related change in the *prostasin *mRNA level (n = 245, age range 26–88) whereas the *PN-1 *mRNA level showed a slight increase with higher age. We were unable to detect any gender-related differences in the *prostasin *or *PN-1 *mRNA expression levels.

### Expression of HAI-1A and HAI-1B during carcinogenesis

**Figure 3:**
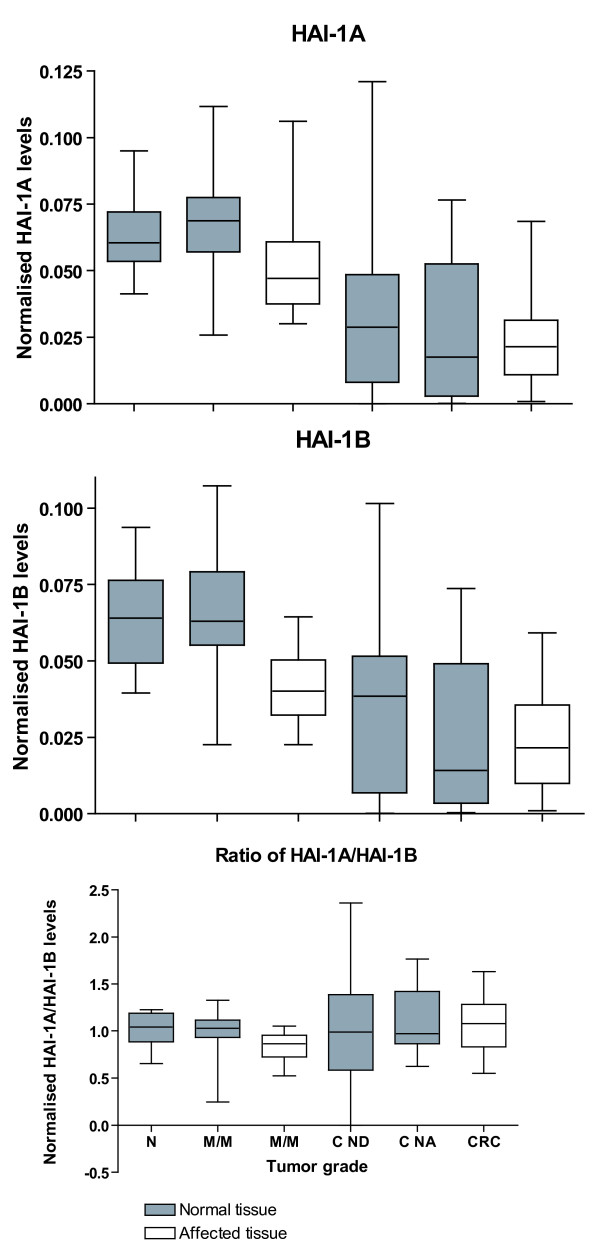
**The levels of *HAI-1A *and *HAI-1B *mRNA were determined by real-time RT-PCR from healthy individuals (N), individuals with mild/moderate dysplasia (M/M) or carcinomas (C)**. Symbols and group designations are the same as in Figure 1. mRNA levels were normalised to the *β-actin *mRNA level. The box shows the interquartile range, the whiskers the range and the median is indicated by a vertical line.

It is possible that prostasin activity is regulated by modulation of the ratio between HAI-1A and HAI-1B as it is not clear whether the two inhibitors have the same kinetic properties. The two *HAI-1 *transcripts differ by the coding region for 16 amino acids. We have previously shown that *HAI-1 *is significantly down-regulated in both normal and affected tissue from individuals with adenomas and carcinomas [34]. In the present study we have investigated a subset of the cohort using two quantitative RT-PCR assays specific for *HAI-1A *and *HAI-1B *respectively. mRNA levels for *HAI-1A *and *HAI-1B *were determined in colon tissue samples from healthy individuals (n = 14), in healthy and affected tissue from individuals with mild/moderate dysplasia (n = 19) and in healthy and tumour tissue from patients with colorectal cancer (n = 24). Figure 3 shows the range and interquartile range of mRNA levels of *HAI-1A, HAI-1B *and the radio of *HAI-1A/HAI-1B *normalised to *β-actin*. The levels of *HAI-1A *and *HAI-1B *mRNA found are virtually identical in all tissues examined with the levels of *HAI-1 *found previously [34]. Both HAI-1A and HAI-1B are thus progressively down-regulated with increasing tumour grade. We thus found no statistically significant difference between the ratios of *HAI-1A/HAI-1B *in the different tissues tested. These data suggest that there is no regulation in the splicing of the *HAI-1 *transcript during colorectal cancer carcinogenesis.

### Localization of prostasin and HAI-1 by immunohistochemistry

**Figure 4:**
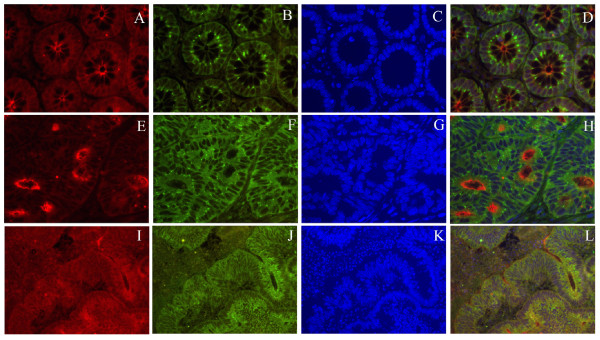
**Prostasin and HAI-1 are located on the apical and the basolateral side, respectively in normal tissue and some colorectal cancer tissue specimens whereas they are co-localized in other colorectal cancer tissues specimens**. Tissue sections of formalin-fixed, paraffin-embedded normal and malignant tissue from colorectal cancer patients was immunohistochemically stained for prostasin (A, E, I) and HAI-1(B, F, J). Histological normal colorectal tissue from a cancer patient (upper panel, A, B, C and D) and two examples of colorectal cancer tissue are shown (middle and lower panel). Nuclei were visualized by DAPI staining (C, G, and K).

Normal and cancerous tissues from five colorectal cancer patients were analysed to investigate the sub-cellular localization of prostasin and HAI-1. Formalin-fixed paraffin-embedded tissue sections were immunohistochemically co-stained for prostasin and HAI-I. In normal colorectal tissue, prostasin was detected mainly on the apical plasma membrane – with only minor staining of the basolateral plasma membrane (Figure 4A and 4D). HAI-1 was found mainly on the basolateral plasma membrane mostly not co-localizing with prostasin (Figure 4B and 4D). This is consistent with previous findings which showing that prostasin is located primarily on the apical plasma membrane [23], and mostly secreted from the apical side of polarized epithelial cells [42]. Likewise previous findings show that HAI-1 is located primarily on the basolateral plasma membrane of a range of polarized epithelial cells [43-46]. In some colorectal cancer tissue specimens we found a similar polarized apical staining of prostasin and basolateral staining of HAI-1 as seen in histologically normal tissue (Figure 4E, F, G, and 4H). In contrast prostasin and HAI-1 were co-localizing in other colorectal cancer tissue specimens all along the cell periphery suggesting that the polarization of the cells has been lost during carcinogenesis (Figure 4L, J, K, and 4L).

## Discussion

In the present study we have determined the mRNA levels of *prostasin, PN-1, HAI-1A *and *HAI-1B *during colorectal cancer carcinogenesis. It has previously been shown that over-expression of prostasin in mammary and prostate cancer cells reduces the invasive properties of cancer cells [23,24] and that high prostasin expression in gastric tumours is associated with longer survival [25]. It could thus be speculated that the transition from severe dysplasia to cancer is accompanied by a transcriptional decrease in prostasin expression and/or a down-regulation of prostasin activity via up regulation of one or more enzymatic inhibitors of prostasin. In the present study we found modest but significantly lower levels of *prostasin *mRNA in mild/moderate dysplasia, severe dysplasia and colorectal cancer tissues combined as compared to corresponding normal tissues from healthy individuals. In a previous study a similar minor but significant down-regulation was seen when comparing normal and affected tissue from gastric cancer patients [25]. Our results do not show any transcriptional down-regulation of *prostasin *accompanying the transition between severe dysplasia and colorectal cancer. However, the mRNA levels of the inhibitor of prostasin, *PN-1 *increase at both the transition between normal tissue and mild/moderate dysplasia and again at the transition between severe dysplasia and colorectal cancer.

For PN-1 to be a physiologically relevant inhibitor of prostasin it is required that PN-1 and prostasin have physical access to each other. PN-1 belongs to the serpin family of secretory proteins. We have previously shown that five other members of the serpin family are secreted to both the apical and the basolateral side of the polarized epithelial cell line, MDCK, albeit at different ratios [47]. Similar results were obtained for the Caco-2 cell line, derived from a human colonic adenocarcinoma (Vogel et al., unpublished results). There are therefore no indications that PN-1 in the extracellular fluid has restricted access to plasma membrane-bound prostasin. Consequently, it is possible that PN-1 plays an important role in down-regulating the enzymatic activity of prostasin in cancer tissue resulting in more invasive cell behaviour.

Prostasin is inhibited not only by PN-1 but also by HAI-1A and HAI-1B. We have previously shown that the level of *HAI-1 *mRNA is decreased by 3-fold during colorectal cancer carcinogenesis using an assay that does not distinguish between *HAI-1A *and *HAI-1B *[34]. In the present study we have shown that *HAI-1A *and *HAI-1B *have expression patterns virtually identical and indistinguishable from the expression pattern of *HAI-1 *previously found [34] and that the ratio of *HAI-1A/HAI-1B *remains the same in all tissues investigated during colorectal cancer carcinogenesis. Therefore, the splicing of *HAI-1 *mRNA is not regulated during carcinogenesis. In the present study we found limited co-localization of prostasin and HAI-1 in normal tissue, as HAI-1 is located mainly on the basolateral plasma membrane and prostasin is located on the apical plasma membrane. This probably does not mean that their interactions is limited due to physical separation as we have previously shown that HAI-1 is transcytosed from the basolateral plasma membrane to the apical plasma membrane [43]. It is thus possible that HAI-1 interacts with prostasin at the apical plasma membrane.

The enzymatic activity of prostasin is influenced both by the levels of PN-1, HAI-1A and HAI-1B and probably also by the expression levels of other as yet undiscovered inhibitors of prostasin. The relative physiological importance of PN-1, HAI-1A and HAI-1B as inhibitors of prostasin is at present unclear. PN-1 is an inhibitor of not only prostasin, but also of uPA, tPA, thrombin, plasmin, trypsin [48] and matriptase [21] and it forms an essentially non-reversible binding to its target protease like most other serpins [49]. Apart from physical accessibilities, the relative abundances of other target proteases may become an important issue as an abundant target for PN-1 may "quench" a large amount of PN-1 by forming a non-reversible complex with it. However, the co-purification of prostasin in complex with PN-1 [49] suggests that PN-1, at least to some degree plays a role as an inhibitor of prostasin *in vivo*. Likewise prostasin-HAI-1B complexes have been purified from a cell line [11], suggesting that it is a physiologically relevant inhibitor of prostasin. Further knowledge about the binding kinetics between the proteases and their inhibitors is needed to clarify the relative importance of PN-1 and HAI-1 isoforms as inhibitors of prostasin.

## Conclusion

Proteases have for a long time been thought to play an important role in the growth of cells both during tissue remodelling and invasive growth. However, the action of proteases may be regulated not necessarily by the expression of the protease but by regulating the expression of its inhibitors. Our data shows that elevated mRNA levels for prostasins inhibitor, *PN-1*, coincides with acquisition of malignant properties, whereas the mRNA level of *prostasin *is relative stable during colorectal cancer carcinogenesis. However, further investigations are necessary to understand the role of prostasin, PN-1, HAI-1A and HAI-1B for invasive growth and malignant progression.

## Competing interests

The authors declare that they have no competing interests.

## Authors' contributions

LV conceived the idea of the study. EHK designed the cohort and collected the samples. LV and EHK extracted the RNA. LV and JB designed and validated primers and probes and performed the statistical analyses. JS-P performed the immunohistochemistry. IMBL evaluated the tissue sections. JS-P, SF and LV prepared the figures. KMT and EKH administrated the KAM study. LV and JS-P wrote the first draft of the manuscript. All authors helped interpret the results, writing the manuscript and read and approved the final version.

## Pre-publication history

The pre-publication history for this paper can be accessed here:

http://www.biomedcentral.com/1471-2407/9/201/prepub
